# Transfer of training from an internal medicine boot camp to the workplace: enhancing and hindering factors

**DOI:** 10.1186/s12909-021-02911-5

**Published:** 2021-09-10

**Authors:** Joanne Kerins, Samantha Eve Smith, Suzanne Anderson Stirling, Judy Wakeling, Victoria Ruth Tallentire

**Affiliations:** 1 Scottish Centre for Simulation and Clinical Human Factors, Stirling Road, Scotland FK5 4WR Larbert, UK; 2 NHS Lothian, Scotland Edinburgh, UK; 3 NHS Education for Scotland, Glasgow, UK

## Abstract

**Background:**

The transfer of training to the workplace is the aim of training interventions. Three primary factors influence transfer: trainee characteristics, training design and work environment influences. Within medical education, the work environment factors influencing transfer of training remain underexplored. Burke and Hutchins’ review of training transfer outlined five work environment influences: opportunity to perform, supervisor/peer support, strategic link, transfer climate and accountability. This study aimed to explore the ways in which work environment factors influence the transfer of training for medical trainees.

**Methods:**

Internal Medicine Training in Scotland includes a three-day boot camp involving simulation-based mastery learning of procedural skills, immersive simulation scenarios and communication workshops. Following ethical approval, trainees were invited to take part in interviews at least three months after following their boot camp. Interviews were semi-structured, anonymised, transcribed verbatim and analysed using template analysis. Member checking interviews were performed to verify findings.

**Results:**

A total of 26 trainees took part in interviews between January 2020 and January 2021. Trainees reported a lack of opportunities to perform procedures in the workplace and challenges relating to the transfer climate, including a lack of appropriate equipment and resistance to change in the workplace. Trainees described a strong sense of personal responsibility to transfer and they felt empowered to change practice in response to the challenges faced.

**Conclusions:**

This study highlights barriers to transfer of training within the clinical workplace including procedural opportunities, a transfer climate with challenging equipment availability and, at times, an unsupportive workplace culture. Trainees are driven by their own sense of personal responsibility; medical educators and healthcare leaders must harness this enthusiasm and take heed of the barriers to assist in the development of strategies to overcome them.

**Supplementary Information:**

The online version contains supplementary material available at 10.1186/s12909-021-02911-5.

## Background

The transfer of training to the workplace is the ultimate aim of training interventions. Transfer of training is defined as “the effective and continuing application, by trainees to their jobs, of the knowledge and skills gained in training” [1]. Training transfer has been investigated in various disciplines over the years, particularly in response to the “transfer problem” highlighted in Baldwin and Ford’s review in 1988 [2]. This is the disparity between training conducted and transfer to the work setting, with training bearing minimal impact on workplace behaviour [2, 3]. The disconnect between educational event and workplace practice has also been recognised within continuing medical education, highlighting a need to better facilitate transfer of training [4]. The three primary factors influencing transfer, first outlined by Baldwin and Ford, are: trainee characteristics, training design and work environment influences [2].

Within medical education, there has been a focus on training design and its potential to influence transfer [5–8]. Simulation training, with an emphasis on experiential and reflective learning, has been described as providing an opportunity to promote self-regulated learning in practice [9]. However, there is a need to better understand the transfer of learning from post-simulation debriefing to real-life situations [10]. Some studies have assessed the influence of learner characteristics, such as personality, finding it independent of transfer [11], and motivation and emotion, finding them to be salient factors for transfer [12, 13]. In addition, retention of skills after educational intervention and ongoing skill maintenance are areas that have received particular attention within medical education although with recognition that they warrant further study [14, 15]. A series of studies in various specialties have found that skills training can be retained well up until around three months [16, 17] and thereafter this can decline with opportunities to perform, booster sessions and simulation-based mastery learning as factors that can improve skill retention [18–22]. Although skill retention is a related concept, training transfer is distinct by virtue of its focus on addressing the ability to transfer skills and training to a different setting or problem.

Many studies assessing skill retention or attempting to assess transfer have done so in controlled simulated environments, rather than in the realities of the clinical workplace [23, 24]. Such approaches neglect to address clinical work environment influences and how these could impact on transfer of learning to the workplace. There have been few attempts to analyse the specific factors that might influence transfer of training [25]. Some studies have explored the acquisition of technical skills within the workplace, recognising the influence of environmental factors and the need for this area to be better understood [26, 27]. Whilst the physical and socio-cultural environment has been considered in workplace learning [28], this area remains underexplored and the breadth of transfer literature in other industries could aid our understanding of such phenomena. Within human resource development literature, Burke and Hutchins performed an integrative review of training transfer in 2007, outlining contributory elements influencing training transfer [29]. The work environment influences include: opportunity to perform; supervisory and peer support; strategic link; transfer climate; and accountability [29]. The definitions of these influences are given in Table 1 and form the conceptual framework for this study [29].

**Table 1:** Work environment influences, as outlined by Burke and Hutchins, with illustrative examples of how they may enhance or hinder training transfer [29]

	Definition	Example
Opportunity to perform	Providing trainees with opportunities to use new learning in their work environment	*Enhancing* *Accounting manager being able to utilise skills learnt in training immediately on return to the workplace* *Hindering* *Airmen having varying opportunities to perform trained tasks due to differences in supervisor attitudes *[30]
Supervisor/Peer support	The social support learners receive from supervisors and peers to use their new skills and knowledge [31]	*Enhancing* *Managers in nuclear power industry networking after training programme has ended and sharing current practice *[32] *Hindering* *Immediate supervisor being unfamiliar with the training content leading to a lack of coaching of new skills on the job*
Strategic link	Alignment of a training program with the strategic direction of an organisation [29]	*Enhancing* *A management development programme including a discussion session with a chief executive to identify ways their managerial approaches contribute to the organisational mission *[33] *Hindering* *Unclear that training intervention supports organisational goals and therefore employees not appreciating the impact of their work on the bigger picture*
Transfer climate	The organisational culture, which projects to its employees varying degrees of a supportive image conducive to the application of new knowledge or skills obtained from training [1, 29, 31, 34]	*Enhancing* *A school providing teachers with new workbooks corresponding to their recent training* *Hindering* *Workers in a fast-food restaurant being ridiculed by more experienced colleagues for using techniques learned in training *[35]
Accountability	The degree to which an organisation, culture, and/or management expects learners to use trained skills on the job and holds them responsible for doing so [29]	*Enhancing* *Managers including goals relating to transfer of training in a formal appraisal process* *Hindering* *Lack of incentive for utilising new skills resulting in reverting back to old habits*

Research into the specific work environment influences listed in Table 1has been dominated by human resource development [29, 30, 32]. Within this context, the opportunity to perform skills learnt is a major influence, with a lack of opportunities being one of the biggest obstacles to transfer of training [30, 36]. This has been noted within the medical education context, for example, internal medical residents finding limited opportunities for joint aspiration and subsequent poor skill retention [18]. This aligns with the accepted concerns around skill decay with lack of practice within medical education [37], but such research does not address the work environment factors that may restrict rehearsal opportunities. Within healthcare, the majority of research in transfer of training is within the nursing education literature [38–40], particularly addressing the importance of supervisor support [41, 42]. The transfer climate within the work environment, or the organisational culture promoting or hindering transfer [31, 34, 35] is thought to be highly influential within healthcare, but there is a lack of confirmatory evidence [39]. The other work environment influences of strategic link, the extent to which the goals of an organisation are aligned with a trainee’s new learning [33], and accountability, holding trainees responsible for using trained skills on the job [29], remain underexplored within the clinical workplace. Understanding these work environment influences in the context of medical education should be of keen interest to those involved in supporting continuing medical education and educational design and delivery, in order to optimise the transfer of skills.

The aim of this qualitative exploratory study was to explore the ways in which work environment factors influence the transfer of training for medical trainees.

## Method

### Context

Internal Medicine Training (IMT) is a three-year training programme for junior doctors in the United Kingdom (UK) wishing to pursue a career in medical specialties. Trainees are eligible to enter IMT after completion of a Bachelor of Medicine and Bachelor of Surgery degree (MBChB; analogous to a combined undergraduate-graduate course), and a subsequent two years of foundation training as junior doctors. In Scotland, a national IMT simulation strategy is embedded within IMT. This involves annual simulation training, including a three-day IMT boot camp within the first year of the training programme.

Between August 2019 and December 2020, the IMT boot camp was delivered to 191 Internal Medicine (IM) trainees in Scotland, in groups of up to 18 trainees. Learning outcomes for boot camp were aligned with the IMT curriculum, grouped into three strands: immersive simulation of acute care situations; mastery learning of procedural skills (lumbar puncture, pleural and ascitic procedures); and communication workshops. Simulation-based mastery learning is an increasingly popular training technique whereby trainees engage in pre-learning and are assessed against pre-determined achievement standards and provided with individualised feedback [43–45]. Trainees also attended an asepsis tutorial to rehearse the preparatory stages of a sterile procedure. All trainees were provided with online pre-learning material in keeping with a flipped classroom method and these online mastery learning resources remained available to them on their return to the workplace. The immersive simulation scenarios involved sepsis, hypoxia, haemorrhagic shock, reduced conscious level, anaphylaxis and cardiac arrest, whereby trainees engaged in a scenario and subsequent debrief focusing on developing non-technical skills. The communication workshops included death and dying, handover and documentation, and interprofessional communication.

### Ethical approval

This study received ethical approval from the NHS Education for Scotland ethics review board (reference number NES/Res/14/20/Med). All participants gave written consent for data collection and the publication of anonymised results. Participants were free to leave the study at any time without giving a reason.

### Data collection

Consenting participants were contacted by email between three and six months after their attendance at boot camp, and invited to an interview. Purposive sampling was utilised whereby trainees were selected from all regions of Scotland, with a mix of genders and age ranges [46].

### Interviews

Qualitative interviews were chosen to allow trainees to provide rich descriptions of their experiences since returning to the workplace [47]. A combination of telephone interviews and interviews via Microsoft Teams were used for the convenience of participants and to comply with COVID-19 lockdown and social distancing measures. Initial interviews took place between 14th January and 9th June 2020. Interviews were conducted by SAS or JW and were subsequently transcribed verbatim. Interviews were semi-structured, centred on the three main themes of the boot camp and the subsequent utilisation of skills in the clinical workplace, whilst permitting deeper exploration of emerging themes. Appendix Adetails the interview guide. A subgroup of six trainees from the initial participants were invited for a further interview to expand upon comments they had made at their initial interview. Four of these trainees agreed and were re-interviewed by JK between 5th and 18th November 2020. The interview primarily focussed on the subcategories of strategic link and accountability, to allow fuller understanding of comments made during iterative data analysis. Subsequently, member checking interviews were conducted by JK between 5th and 20th January 2021 with the second cohort of IM trainees, where the initial study findings were presented and discussed to verify and refine the findings [48]. This process aimed to enhance the trustworthiness of the data. Interviews were completed when no new subthemes emerged and it was deemed that saturation had been reached [49].

### Data analysis

Interview transcripts were independently analysed by JK, SES and VT using template analysis [50]. In template analysis, a template based on prior research and theoretical perspectives is applied and the initial template may be modified by the data with new codes added inductively [50]. Analysis commenced in parallel with continued data collection in order to facilitate the deeper exploration of emerging themes with subsequent participants. This subgroup of the research team (JK, SES and VT) met on a regular basis, discussed each category of the framework in detail and compared coding which informed iterations to the interview schedule throughout the process.

The work environment influences subsection from Burke and Hutchins’ review of training transfer (Table 1) was utilised as the initial coding framework [29]. Disagreements on coding were discussed with reference to Burke and Hutchins’ literature review, with final decisions on analysis made by JK [14]. The resultant framework is therefore her conceptualisation of the framework produced by the interactions between JK, the research participants and her co-researchers.

#### Reflexivity

It is recognised through the constructivist nature of the study that ideas are co-constructed between participants and researchers, and that the researchers prior clinical and educational experiences will influence the findings and their interpretation. The researchers involved in this study brought a breadth of experience with a mixture of research (SAS and JW) and clinical (JK, SES and VT) backgrounds: SAS and JW are specialist research leads at NHS Education for Scotland with extensive qualitative research experience; JK is an acute medicine doctor with eight years of postgraduate clinical training and experience of medical education research; SES is a general practitioner with a special interest and doctoral degree in medical education; VT is a consultant in acute medicine, a simulation educator and a post-doctoral medical education researcher. The initial interviews were conducted by SAS and JW who had not been present at the boot camp and so were deemed impartial when enquiring about the trainees’ reflections relating to transfer.

## Results

A total of 26 trainees took part in interviews consisting of 16 initial interviews, four re-interviews and ten member checking interviews, each lasting between 15 and 35 min (average of 21 min 43 s). Participants included 11 males, 14 females and one trainee who preferred not to categorise their gender. Participants were aged between 24 and 35. They were from the West, South East and North regions of Scotland and included participants from all six boot camps from the initial 2019-20 cohort.

**Fig. 1:**
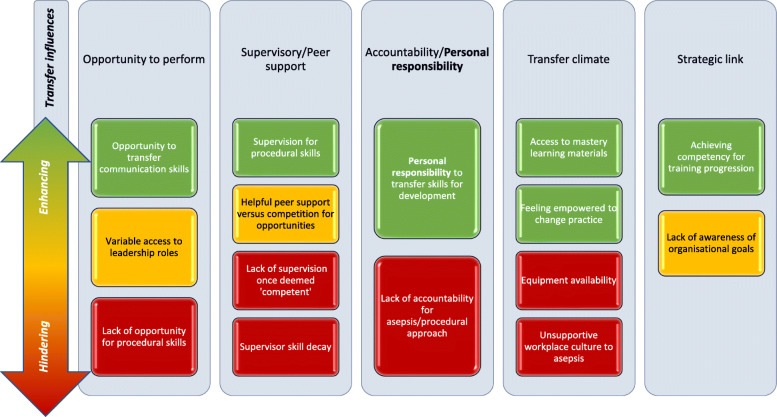
Amended workplace influences framework as applied to the context of transfer from an internal medicine trainee boot camp

The work environment influences from the initial coding framework (utilising the subcategories described by Burke and Hutchins[29]) resonated with trainees as salient factors relating to transfer of training to the clinical workplace. There was evidence of how these factors promoted or inhibited transfer of their training with amendments to the original framework, as illustrated in Fig. 1, with example quotes in Table 2.

**Table 2:** Work environment influences on transfer of training for internal medical trainees

	Enhancing transfer	Hindering transfer
Opportunity to perform	**Opportunities to transfer communication skills** ‘*I think there were just certain phrases and ways of having a conversation that I definitely put into my vocabulary for having these conversations. I definitely found myself using them when I have been having those conversations*.’ (Trainee 9) **Opportunities to transfer acute care skills** *‘I have had a few asthma attacks I have had to phone intensive care about, so I have been more confident with that and the need to get someone else involved.’* (Trainee 6)	**Lack of procedural opportunities** *‘I still have not yet been able to do any of those procedures because it has just not come up where I am working just now’* (Trainee 3) *‘Because I’m on medicine for the elderly and…there’s not that much opportunity for chest drains.’* (Trainee 4) ‘*I have done some lumbar punctures, not so much chest drains and ascitic stuff, but that is just because of the jobs I have been on*.’ (Trainee 17)
Supervisory/Peer support	**Supervisor availability** *‘I had the oncology registrar looking over my shoulder being my assistant at the time, but I managed by myself without any input and I was really confident that I was doing the right thing.’* (Trainee 12) **Peer support** *‘There is a bit of bouncing ideas off each other when there is nobody more senior about.’* (Trainee 20)	**Lack of supervision once deemed competent** ‘*I have not received any feedback in the workplace…If you are competent with a procedure…you tend not to be directly supervised in the workplace*.’ (Trainee 1) **Supervisor skill decay** *‘A lot of good registrars have said that they’re not trained to use them [atraumatic lumbar puncture needles] and then obviously that’s the way we should be doing LPs [lumbar punctures], that’s obviously a bit tricky’* (Trainee 25)
Strategic link	**Link to IMT curriculum** *‘I wouldn’t really know what the organisational goals are, but I think it’s very clear what the IMT goals are, there’s no uncertainty about what you need to do there, and I think that the course definitely aligned with that’* (Trainee 24)	
Transfer climate	**Feeling empowered to change practice** *‘Unfortunately, that [aseptic approach] wasn’t happening in the hospitals but as of now we lead by example.’* (Trainee 10) *‘if you are coming up against that kind of culture, you can say “this is what the evidence says to do, and we practise evidence-based medicine.”’* (Trainee 24) **Mastery learning resources** *‘to have access to those materials thereafter because of all the learning packs provided good revision…I can look through them just before I do a procedure just as a point of reference.’* (Trainee 7)	**Lack of equipment** *‘It can be difficult to find things like sterile gloves on the ward. So that’s the challenge - getting the right equipment.’* (Trainee 4) *‘Even just getting the right gauge needles for lumbar puncture or the right dressing pack, that really simple stuff, certainly the wards I had worked on have really struggled to provide all of that in a consistent way.’* (Trainee 8) **Resistance to change** *‘I don’t think a lot of people are very interested in helping you find gowns and stuff to do lumbar punctures.’* (Trainee 2) *‘’Oh, why are you doing that?’, even just with a lumbar puncture I try and find an introducer needle, and they are like ‘you don’t need an introducer needle, that’s not what you need’. I feel like the attitude of people has been like ‘oh, it doesn’t really matter you know.’’* (Trainee 17)
Accountability	**ePortfolio** *‘I think ePortfolio is a big driver. It is one of the things that helps us to keep track with what we are doing.’* (Trainee 22) **Personal responsibility** ‘*Thinking ahead, I guess if you are going to be the registrar on call and you are the one that it is going to be escalated to, you want to be able to be competent and confident in procedures that are quite common, that you might have to do.*’ (Trainee 21) *‘I want to be a better doctor…I am keen to do my job well.’* (Trainee 19) *‘You have got to be driven in yourself to do it’* (Trainee 17)	**Lack of accountability in the work environment** - **Supervisor ambivalence**: *‘My supervisor was both fed-up with me and impressed by the fact that I wanted to lay out all my equipment in the order I was using it’* (Trainee 9) *‘I have experienced that, where people who supervise you say “you don’t need a full gown and things like that” or “I don’t do it that way.”’* (Trainee 21)

### Opportunity to perform

The opportunity to perform procedural skills was identified by some trainees as pivotal to the transfer of their new skills: ‘I had done a few at boot camp and then went back and did another few under supervision and now I am generally working independently.’ (Trainee 2). However, many trainees found a lack of opportunity to perform certain practical procedures in the workplace frustrating. Opportunities were thought to be based on a combination of specialty post, colleagues and luck: ‘Even if you are in a job where you might get the opportunity, so much of it is: Is it a patient you are looking after? Who is on with you? So much of it is luck I think.’ (Trainee 17). Trainees expressed that ‘it is a shame because that leaves a big gap between doing the teaching and the doing the procedures.’ (Trainee 9). In particular trainees did not have the opportunity to perform pleural procedures, finding it ‘nigh on impossible’ (Trainee 23) if not on a respiratory placement. This situation was viewed as ‘a vicious cycle, that non-respiratory people are not that confident in doing them [pleural procedures] and therefore respiratory do them, which means that non-respiratory do it less.’ (Trainee 23).

Dealing with acute medical emergencies and being able to draw on the non-technical skills covered at boot camp, was more easily accessible for trainees. For example, taking on leadership roles in similar scenarios:

‘…the discussion on how best to be a leader in that situation…to just step back and have an overview and a calm approach of the situation- I try and be [at] the end of the bed, kind of, overview.’ (Trainee 18).

When leading acute situations, trainees described opportunities to utilise the training in escalating care, anticipating and future planning: ‘I tend to do that quite a lot now even if the patient does not need to go to intensive care but is unwell, that intensive care people should be aware at an early stage in case things deteriorate further.’ (Trainee 3). Although most trainees had access to leadership opportunities, some did find that they were not in roles where they were leading acute emergencies. Trainees found the opportunity to transfer some aspects of the reflective practice incorporated within the simulation debriefs at boot camp to the workplace: ‘It changed me by allowing me to reflect upon emergency simulation and thinking how I approach that situation differently in the future. Would I have done that had I not gone to the IMT training? I think healthy reflective process started following the boot camp.’ (Trainee 1).

The opportunity to transfer training from the communication workshops was the most readily accessible for trainees when returning to the workplace. In particular, the opportunity to put into practice the training from the ‘death and dying’ workshop was noted by many trainees: ‘There have been a number of times a patient has been unwell, and I have had to speak to their families, and I am using the phrase that we got taught: “they are sick enough to die”.’ (Trainee 6) Trainees felt that challenging interactions were part of the ‘everyday things that we do’ (Trainee 17) including interprofessional communication:

‘I think after boot camp I have been a bit more confident about how I speak to colleagues or just a bit more understanding. Reflecting on how I communicate with colleagues, reflecting on how I approach difficult scenarios with colleagues…So, it has changed the way I work as a colleague.’ (Trainee 15).

The 'handover and documentation workshop' covers how to manage clinical error, which some trainees were able to transfer into practice: ‘An FY1 [more junior doctor] had looked at the wrong scan and the patient had been transferred to another hospital and I was able to help her a wee bit with that because of our conversation [at boot camp]…[and] support her in telling the family and letting the consultant know.’ (Trainee 2). Overall, the opportunities trainees had to perform and rehearse their enhanced non-technical and communication skills had a positive influence on transfer, but finding opportunities to perform procedural skills was more challenging which hindered transfer for many trainees.

### Supervisor/Peer support

Trainees found supervisor support beneficial in promoting transfer, particularly of procedural skills. They felt ‘very well supported in terms of needing supervision for things, because you are very comfortable in saying no… I don’t think it’s okay to do a procedure if you can’t get the appropriate supervision.’ (Trainee 5). However, the trainees expressed concern that there is a lack of supervision once they are deemed ‘competent’:

‘I think there is a barrier there to seeking supervision after the point that someone has said that you’re independent to do it …It is very easy then to get into slightly bad habits or your let some of the techniques slip a little bit.’ (Trainee 8).

Although trainees felt that ongoing supervision, even once deemed competent, would be beneficial, they recognised it is not always realistic: ‘There are not enough doctors to all watch each other do skills all the time. It is just not practical to do that. Once you are competent you have to go off on your own and do it yourself.’ (Trainee 17) It was also recognised that supervisors may not be familiar with the current method being taught at boot camp, as trainees commented they may be ‘directly supervised by a consultant who possibly learnt to do lumbar punctures 30 years ago’ (Trainee 18) and that ‘consultants are maybe a bit far removed.’ (Trainee 24) In response to a lack of supervisor awareness of current guidance, trainees felt empowered to transfer skills in the way they had been taught at boot camp:

‘“Oh, we don’t normally do it like that”, but once I have explained the kind of teaching I have had, there has not been any problem with me doing it like that.’ (Trainee 18).

However, some noted that they might find this situation difficult depending on the seniority of the supervisor and the inherent power dynamic: ‘I kept trying to find one [atraumatic lumbar puncture needle] because I am more comfortable with doing it this way. I say “I appreciate that is the way that you have done it, but this is the way I am more comfortable doing it.” It was easy in that situation because it was with somebody more my level. I would imagine if you had a consultant with you, you would probably be like “okay, we will do what you say.”’ (Trainee 17).

The challenges of accessing opportunities for transfer of procedural skills can create competition between peers: ‘I have been in a situation a couple of times where you were with another IMT and they are like, “Oh, do you want to do this one and I will do the next one”…It is kind of a competition thing then, who gets to take this one?’ (Trainee 17) However, in relation to the non-technical and communication skills covered at boot camp, trainees found peer support beneficial: ‘I think we quite like to bounce ideas off each other to be honest. The environment here is more, they like to have buddy IMTs…to have discussions surrounding things or how you manage a patient or how you get the procedure done.’ (Trainee 22).

### Strategic link

Strategic link refers to the extent to which training aligns to the goals of an organisation; for this cohort the responsible organisation is NHS Education for Scotland (NES). However, trainees were unaware of the specific goals of NES and so this was not an influential factor in their transfer of training. When prompted with the NES strategy and key areas of focus[51], there was some recognition that the boot camp training aligned well with NES strategic aims. For example, trainees appreciated that the aim of having a ‘trained and compassionate workforce definitely would be in line with that [boot camp].’ (Trainee 17) Trainees felt that the training aligned to the ‘IMT goals’ (Trainee 24) well but on a wider, organisational level, strategic link was not a significant factor in influencing transfer of training to the workplace for this cohort.

### Transfer climate

The transfer climate was recognised as a significant factor influencing transfer of training from the boot camp to clinical practice. Regarding transferring procedural skills, a lack of equipment availability was noted to be a challenge:

‘You cannot find any on the ward, you have to traipse round different wards because equipment is in various different places and not set up and formalised.’ (Trainee 21).

In particular, trainees emphasised the difficulties of putting into practice the approach to asepsis they had been taught for procedures during the boot camp. There was often a lack of equipment: ‘I think it can be quite difficult to find proper gowns, perhaps outside of a theatre setting’ (Trainee 2), but they also expressed a sense that the workplace culture relating to asepsis was unsupportive; ‘I get some weird looks when I go looking for gowns.’ (Trainee 14). Trainees admitted that this lack of support proved to be an obstacle to transferring training from the boot camp to their workplace. Some trainees reverted back to previous, out-dated practice: ‘So it ended up being just an apron and a pair of sterile gloves’ (Trainee 12), admitting that ‘you might not be as thorough as you should be, especially when certain equipment isn’t available.’ (Trainee 10). In contrast, some trainees felt empowered to be a force of change in the workplace: ‘I think when it comes to ourselves, having been through the boot camp, we can choose to do [things] the way we think is the best or the way we have been taught’ (Trainee 22).

‘It reinforces a time for change and that we are probably the generation to make that change happen.’ (Trainee 5).

Trainees reflected on the benefits of ongoing access to the online mastery learning resources for procedural skills in aiding their transfer of such skills to the workplace: ‘I think the mastery learning tool was a great resource as a refresher.’ (Trainee 18). The accessibility of training materials allowed trainees to transfer the mastery procedural approach, even when there had been a gap between the boot camp and performing the procedure, thereby mitigating, to some extent, the lack of opportunity described earlier.

### Accountability

In order to progress to the next stage of their training, trainees are expected to achieve competency in the procedures rehearsed at boot camp. However, trainees are not specifically held accountable for transferring these skills: ‘All we needed for IMT was to sign off at the boot camp level – I don’t think we needed to go beyond that. So it was more just for my own learning and interest and feeling like I should take the opportunity.’ (Trainee 2). From the findings relating to transfer climate described above, there was evidence of a lack of accountability by supervisors for procedural asepsis in the workplace. IM trainees have an online portfolio (ePortfolio) to document their progression and an Annual Review of Competency Progression (ARCP). The alignment of the boot camp with the IMT curriculum promoted transfer for trainees who were ‘very portfolio driven’ (Trainee 18) and recognised ‘the pressure of getting signed off for the portfolio and meeting the ARCP requirements.’ (Trainee 11).

However, a more prominent influence on trainees’ transfer was their own ‘personal development’ (Trainee 20) and trainees held themselves accountable: ‘I forget about the portfolio…the main incentive is my own development and wanting to get better at things.’ (Trainee 17). They described ‘that sense that you are progressing, seeing that you are able to manage these complicated unwell patients reinforces that sense of professional development and growth and competency.’ (Trainee 8). They anticipated the need to hone skills for when they become medical registrars and are often the most senior medical doctor on site out of hours:

‘If you imagine yourself being in a small hospital and there is nobody else that could do certain things, it is really quite handy to have someone who can do a chest drain or do a lumbar puncture, you really become a valuable asset in terms of getting really useful stuff done.’ (Trainee 20).

There was evidence of future planning and holding themselves accountable in light of the roles that they will take on in the next few years. A sense of personal responsibility proved to be a stronger influence on transfer than the idea of others in the workplace holding them accountable.

### Member checking interviews

The results of the member checking interviews corroborated the findings from the initial interviews; application of the transfer framework in this context resonated with the trainees’ experiences. In considering equipment availability for full asepsis, there were some improvements as trainees acknowledged that ‘the world has changed a bit’ (Trainee 13) due to the COVID-19 pandemic and that ‘there are masks everywhere, but previously it was hard to find a facial mask on the ward.’ (Trainee 11). In particular, trainees agreed that strategic link was not a prominent factor for transfer from an organisational goals perspective.

## Discussion

This study explored the factors influencing the transfer of training from a three-day boot camp for internal medicine trainees in Scotland. Burke and Hutchins’ work environment influences category, and subcategories therein, were used as a conceptual framework to analyse the data, allowing us to explore how the clinical workplace itself influences the transfer of training[29]. Although there is a breadth of literature on skill retention, this study assesses the concept of transfer of training, moving beyond the classroom or simulation centre to the clinical workplace, and provides an in-depth analysis of the workplace influences that can impact this.

The most striking finding from this study was the work environment influences that hindered the transfer of procedural skills, particularly the transfer climate. Trainees described a transfer climate with poor access to equipment and a challenging workplace culture towards change, especially regarding an aseptic approach to invasive procedures. It was recognised that, opportunistically due to the COVID-19 pandemic, the availability of personal protective equipment on medical wards has improved access to the equipment required to perform procedures with full asepsis. However, the lack of equipment availability and supportive culture heralds a more challenging issue around reluctance to change in the clinical workplace. For example, there has long been evidence to support the use of atraumatic needles for lumbar puncture and yet a widespread failure to adopt this change persists[52]. Resistance to change is well recognised in healthcare with numerous causative factors identified including: embedded routines; leader inaction and cynicism[53]; and inadequate efforts to keep up with nationally recognised standards[54]. Being met by opposition or cynicism on attempting to transfer training to the workplace was also highlighted in a study of nurses in China, undermining transfer attempts[40]. The ward climate has also been emphasised as a reason for resistance to change practice in mental health wards[55]. This study has highlighted the particular challenges faced by trainees when training conflicts with current culture and accepted practice in the workplace. An encouraging finding in this study was that many trainees felt empowered to be able to counter any cynicism, reinforcing in their minds that it was ‘a time for change’. For some trainees, it was also relatively easy to overcome obstructive individuals once they ‘explained the kind of teaching I have’.

In Burke and Hutchins’ review they found that the variables of strategic link and accountability had the least support in the literature relating to influence on transfer[29]. Our findings partially echoed this; strategic link was not an influential factor for transfer. Interestingly, accountability did appear to be an important factor for transfer in our context. Although the transfer of the skills from the boot camp is not specifically prescribed or compulsory for the trainees, they shared a sense of responsibility to do so. Their sense of accountability related to wanting to ‘be a better doctor’ which also links to the training transfer category of learner characteristics and their motivation to transfer[29]. Although we have included this new subtheme within the category of accountability, this is in contrast with the initial coding framework where accountability focussed on others’ expectations, rather than holding oneself accountable. More recent work on accountability has broadened the appreciation of personal responsibility in influencing transfer, and particularly relevant in this context is the concept of role responsibility[56, 57]. Medical trainees expressed a sense of personal responsibility for putting skills into practice to support personal development, conscious of their future roles. Trainees highlighted the need ‘to be driven in yourself to do it’ and whilst this is helpful in promoting transfer, medical educators must take heed of the barriers highlighted in this study, including opportunities to perform and supervisor support, in order to facilitate transfer. There will always be challenges within the complexities of the clinical workplace, but rather than relying on trainees’ sense of personal responsibility, this study highlights specific areas requiring attention to improve the disconnect between the classroom or simulation centre and the workplace.

Training interventions should not exist in a bubble, and efforts should be made to improve the ties between the training environment and clinical workplace. As Mironoff highlighted in 1988, training ‘cannot create new behaviour for an environment that will not support it’[58]. To improve the transfer climate, engaging with trainee supervisors and departments, and communicating up-to-date guidance and equipment requirements for procedures, should be the foci going forward. There is a need for supervisors to be mindful of their own skill decay and to empower trainees to take opportunities to perform procedures and act in leadership roles with supervision where possible. This study highlights the realities of procedural exposure and, as a result, further training has been implemented for this cohort in the subsequent year, to revisit procedural skills that they have not had the opportunity to perform in clinical practice. As a collective healthcare community, we must be aware of a pervasive reluctance to change in the clinical workplace, and take responsibility to encourage best practice and remain open-minded to updates. In doing so, we may harness the enthusiasm for improvement expressed by trainees in this study to facilitate positive change and enhance safety within the workplace.

### Strengths and limitations

This study accessed a national sample of trainees over a two-year period providing deep insights into the mechanisms of training transfer across Scotland. It explored the environmental factors of transfer that are not well evaluated in medical education, although did so solely from the perspective of trainees and the results are therefore limited to their subjective perception of transfer. Trainees volunteered to take part in the interview process resulting in possible self-selection bias whereby trainees with particularly strong feelings towards the boot camp may have been more likely to volunteer. We explicitly asked for trainees to provide honest accounts of their experiences and ensured confidentiality. However, despite JK having no supervisory role for the trainees involved, her clinical role as a medical registrar and involvement in the boot camp may have influenced their accounts of transfer. Although trainees reflected on the transfer of all aspects of the boot camp, the fact that performance of procedural skills is more tangible to recount may explain the predominance of procedure-related issues in the results. It is also possible that trainees attended additional courses after boot camp that could have influenced their transfer of training experiences. However, particularly for the second cohort who attended boot camp during the COVID-19 pandemic, this was deemed unlikely to have influenced the results significantly.

The use of a framework from another industry provides a useful lens to facilitate insight into the phenomenon of transfer of training to the clinical environment. However, we must be cognisant that it was not developed in a medical context and remain open-minded to other workplace influences. The use of a conceptual framework aimed to heighten transferability of the research findings and, although the context described here is an internal medicine boot camp in the UK, the intervention design includes simulation training with debriefing and simulation-based mastery learning of procedural skills, both of which are used internationally.

### Further research

Given that the study is solely from the perspective of trainees, investigating supervisor, departmental or organisational perspectives would also be beneficial in exploring work environment issues. Regarding the procedural exposure considerations highlighted by this study, further work could investigate whether the boot camp training provided, and IMT curriculum reflects, the realities and requirements of clinical practice, in order to support constructive alignment of curricula and the clinical environment. Further research could focus on the other categories known to influence transfer, in particular the influence of learner characteristics, including the motivation of trainees. In addition, action research aimed at changing the transfer climate or supervisor attitudes to better facilitate transfer of learning would be helpful. Given this study focuses on transfer from a stand-alone boot camp, studies investigating transfer during or following longitudinal styles of training would be helpful.

## Conclusions

This study utilised a pre-existing conceptual framework for training transfer, to explore the factors influencing transfer of a new training intervention for internal medical trainees in Scotland. In doing so, it has shed light on specific barriers that hinder transfer in the workplace including procedural opportunities, equipment availability and unsupportive workplace culture. Addressing the work environment barriers highlighted, coupled with ongoing boot camp training to promote best practice, should enhance transfer and, in turn, patient safety. This study reinforces the notion that our role as medical educators extends beyond the classroom and we must consider workplace factors to improve the likelihood of training successfully influencing clinical practice.

## Supplementary Information

**Additional file 1:**

## Data Availability

The data analysed during the current study are not publicly available in the interest of participant privacy but are available from the corresponding author on reasonable request

## Declarations

### Authors' information

Dr Joanne Kerins is an Acute Medical Registrar in NHS Greater Glasgow and Clyde and a Simulation Research Fellow at Scottish Centre for Simulation and Clinical Human Factors (SCSChf), Larbert, United Kingdom (UK).

Dr Samantha Eve Smith is a General Practitioner in Edinburgh, UK.

Ms Suzanne Anderson Stirling is a Specialist Research Lead with NHS Education for Scotland (NES), Glasgow. UK.

Dr Judy Wakeling is a Specialist Research Lead at NHS Education for Scotland, Glasgow, UK. Dr Vicky Tallentire is an Educational Co-ordinator at SCSChf, a consultant in Acute Medicine in NHS Lothian and Associate Postgraduate Dean for NES.
