# Supplementary material for: Transfer of training from an internal medicine boot camp to the workplace: enhancing and hindering factors
Source: BMC Med Educ. 2021 Sep 10;21:485. doi: 10.1186/s12909-021-02911-5 (PMC8428956; doi:10.1186/s12909-021-02911-5)
Supplement: Supplementary file 1 — Additional file 1: [file 12909_2021_2911_MOESM1_ESM.docx]

**Appendix A**

**Interview schedule from study exploring transfer of training of internal medicine trainees in Scotland 2020-21**

1. What do you remember about the boot camp? Has this changed your real-life practice? Have you encountered any challenges in the implementation of this training?
2. Since boot camp, do you think you have used any of the skills in practice? Have you had the opportunity to perform any of the skills you rehearsed at BC in your clinical practice? If yes, which ones and how did that come about? If no, why do you think not? What has prevented you?
3. What support have you received to use the new skills you learnt at BC in your clinical practice? To what extent have you been supported to use your skills by your supervisors? Have your peers supported you using your newfound skills in any way?
4. Could you tell me the stated aims of your organisation? Do you think that the aims of boot camp were linked to the aims of your organisation or the department where you work? Do you think the aims are aligned or misaligned (and in what way)?
5. What cues have prompted you to use the skills you learned at BC in the workplace? Have you (or anyone else) set yourself goals for using the skills in practice? Do you think the climate in the workplace is supportive of you using what you learnt at BC in your clinical practice? Is yes, how; if no, why not?
6. Are there incentives to use new skills? What are the consequences for you personally of having developed new skills at BC? Did you feel it was expected that you would use the skills you learnt at BC in the clinical workplace? If yes, what made you feel that? If no, why do you think that was? Do you feel the e-portfolio helps to define what is expected of you as an IM1? What motivates you to transfer training?
